# Intrauterine Transmission of SARS-CoV-2

**DOI:** 10.3201/eid2702.203824

**Published:** 2021-02

**Authors:** Emanuele T. S. Stonoga, Laura de Almeida Lanzoni, Patricia Zadorosnei Rebutini, André Luiz Permegiani de Oliveira, Jullie Anne Chiste, Cyllian Arias Fugaça, Daniele M. M. Prá, Ana Paula Percicote, Andrea Rossoni, Meri Bordignon Nogueira, Lucia de Noronha, Sonia Mara Raboni

**Affiliations:** Hospital de Clínicas da Universidade Federal do Paraná, Parana, Brazil (E.T.S. Stonoga, L.A. Lanzoni, J.A. Chiste, C.A. Fugaça, M.B. Nogueira);; Pontifícia Universidade Católica do Paraná, Parana (P.Z. Rebutini, A.L.P. Oliveira, D.M. Marani Prá, L. de Noronha);; Universidade Federal do Paraná, Parana (A.P. Percicote, A. Rossoni, S.M. Raboni)

**Keywords:** SARS-CoV-2, COVID-19, stillbirth, placenta, autopsy, congenital transmission, RT-PCR, swabs, saliva, respiratory infections, severe acute respiratory syndrome coronavirus 2, 2019 novel coronavirus disease, coronavirus disease, zoonoses, viruses, coronavirus, diagnosis, testing, upper respiratory swab samples, pregnancy, Brazil, intrauterine transmission

## Abstract

We documented fetal death associated with intrauterine transmission of severe acute respiratory syndrome coronavirus 2. We found chronic histiocytic intervillositis, maternal and fetal vascular malperfusion, microglial hyperplasia, and lymphocytic infiltrate in muscle in the placenta and fetal tissue. Placenta and umbilical cord blood tested positive for the virus by PCR, confirming transplacental transmission.

A woman 42 years of age at 27 weeks’ gestation sought treatment at Hospital de Clínicas da Universidade Federal do Paraná, Parana, Brazil, for symptoms of coronavirus disease (COVID-19). Dyspnea, dry cough, high temperature (38.5°C), anosmia, nausea, vomiting, and diarrhea had developed 2 days before hospitalization. At admission, we collected a nasopharyngeal swab sample and tested it for severe acute respiratory syndrome coronavirus 2 (SARS-CoV-2) and rhinovirus by reverse transcription PCR (RT-PCR) (XGEN MASTER COVID-19 Kit; Mobius Life Science, Inc, https://mobiuslife.com.br) (Appendix). The sample tested positive for both viruses. We prescribed azithromycin, oseltamivir, prophylactic enoxaparin, and corticosteroids for fetal lung maturation. A chest computed tomography scan revealed bilateral ground glass opacities and interlobular septal thickening. After 4 days, the patient needed ventilatory and hemodynamic support.

The patient’s prenatal care had been uneventful. She had undergone routine tests and ultrasound scans; the most recent had been at 25 weeks’ gestation. Her medical history included a previous pregnancy complicated by hypertension that resolved with delivery. The current pregnancy was her seventh; she previously had delivered 3 children and had 2 abortions and 1 ectopic pregnancy.

Six days after admission, obstetric ultrasound demonstrated a single intrauterine pregnancy. The fetus was in a transverse position with shoulder presentation; the ultrasound showed reduced amniotic fluid volume and absence of fetal movements and heart rate. Because misoprostol failed to induce labor, we conducted a cesarean delivery. The fetus was stillborn. Immediately after delivery, we used an aseptic technique to collect samples of amniotic fluid (before amniotic membranes ruptured), umbilical cord blood, placental membranes, and cotyledon fragments (Table).

**Table:** Results of PCR for severe acute respiratory syndrome coronavirus 2 in a pregnant woman and fetus, Brazil, 2020*

Sample	Day	Cycle threshold†		
		ORF1ab	N	RNaseP‡
Maternal nasopharyngeal swab sample	0	21.0	24.0	23.0
Maternal nasopharyngeal swab sample	4	20.9	24.8	29.9
Umbilical cord blood	8	31.9	30.3	27.0
Placenta§	8	24.5	25.5	25.6
Fetal liver	9	Undetectable	Undetectable	29.0
Fetal spleen	9	Undetectable	Undetectable	27.8
Fetal lungs	9	Undetectable	Undetectable	25.7
Fetal central nervous system	9	Undetectable	Undetectable	29.4
Fetal skeletal muscle	9	Undetectable	Undetectable	26.5
Fetal heart	9	Undetectable	Undetectable	26.5
Fetal ovary	9	Undetectable	Undetectable	25.4

*PCR conducted using XGEN MASTER COVID-19 Kit (Mobius Life Science, Inc, https://mobiuslife.com.br). N, nucleocapsid protein gene; ORF, open reading frame. †Cycle threshold value is considered positive if both viral genes are <38. ‡PCR is selective for human RNaseP gene as a control for sample integrity. §Insufficient sample.

We obtained informed written consent for fetal autopsy, placental grossing, and histologic examination. External examination showed a female concept with skin discoloration and moderate peeling; the fetus had gestational age of »28 weeks and weighed 1,020 g (50th percentile). Internal examination revealed red serous effusions in the chest and abdomen and petechial hemorrhage in the heart and lungs. We conducted evisceration using the Letulle method and separated the organs into functional groups. We noted hepatic discoloration and friability and lung and kidney hypoplasia (both <5th percentile). We did not identify other macroscopic abnormalities.

The placental disc was round and had tan and glistening membranes peripherally attached. The umbilical cord had 3 vessels; it was 28 cm long, inserted eccentrically, and under coiled. The fetal surface was gray with normal chorionic plate vessels. The trimmed placental disc weighed 135 g and measured 12 × 12 cm (<3rd percentile) (Appendix). We collected additional samples of fetal liver, spleen, lung, central nervous system tissue, ovary, and muscle for RT-PCR (Table). Tissue samples were fixed in 10% buffered formalin, routinely processed, stained in hematoxylin and eosin, and underwent immunohistochemical staining using CD68 antibodies (Figure; Appendix).

**Figure:**
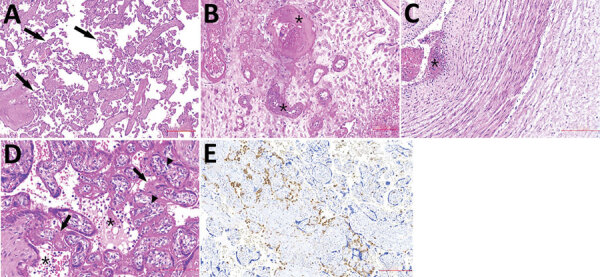
Histologic sections from the placenta of stillborn fetus of a woman with severe acute respiratory syndrome coronavirus 2 infection, Brazil, 2020. Tissue stained with hematoxylin and eosin. A) Placenta shows accelerated villous maturation with increase in syncytial knots. Black arrows indicate small or short hyper mature villi. B) Membranes and basal decidua show decidual arteriopathy, including fibrinoid necrosis with foam cells, mural hypertrophy, absence of spiral artery remodeling, and arterial thrombosis associated with decidual infarct. Asterisks (*) indicate fibrinoid necrosis. C) The umbilical cord shows subendothelial edema and nonocclusive arterial thrombosis, which was also focally observed in a chorionic plate and stem vessels. Asterisks (*) indicate arterial thrombosis. D–E) Photomicrographs show diffuse perivillous fibrin deposition associated with multifocal mononuclear inflammatory infiltrate in the intervillous space and occasional intervillous thrombi. Black arrows indicate fibrin deposition; asterisks (*) indicate mononuclear infiltrate; arrowheads indicate increase in number of Hofbauer cells. E) Immunohistochemical assay using CD68 antibodies highlights histiocyte infiltrate in paraffin-embedded samples (KP1 Clone; Biocare Medical LLC, https://biocare.net).

Few reports have described the effects of SARS-CoV-2 infection in utero; because pathogen detection requires multiple samples, it has been difficult to characterize congenital infection (*1*,*2*). According to Shah et al. (*3*), congenital SARS-CoV-2 infection can be confirmed by PCR of placental tissue. We detected SARS-CoV-2 RNA in cotyledon samples, membranes, and umbilical cord blood aspirate, suggesting a breakdown of the placental barrier and fetal intrauterine viremia. We used immunohistochemical staining with CD68 antibodies to identify multifocal chronic histiocytic intervillositis in the placenta (Figure, panels D, E). This condition was also described in other pregnant women with COVID-19 (*4*,*5*). We also noted microglial hyperplasia, mild lymphocytic infiltrate, and edema in skeletal muscle (Appendix). These findings might suggest infection. However, all fetal tissue samples tested negative for SARS-CoV-2 RNA (Table). Other findings might have been caused by intrauterine asphyxia (Appendix).

COVID-19 is associated with cytokine storm, an exaggerated inflammatory response that is usually indicative of disease severity (*6*). Excessive inflammation could cause endothelial damage and disrupt the coagulation system; some evidence suggests that thrombotic and microvascular injury might affect manifestations of COVID-19 (*7*,*8*). We noted severe maternal vascular malperfusion injuries in the placenta, including substantial recent infarcts, decidual vasculopathy, accelerated villous maturation, and low placental weight. Similar findings are often observed in placentas from women with hypertensive disorders and have been associated with oligohydramnios, preterm birth, and stillbirth. Although the patient’s blood pressure was within reference limits, her age and history of gestational hypertension are risk factors for such alterations and the probable cause of placental insufficiency and fetal demise (*9*,*10*). We also observed multifocal small intervillous thrombi and focal thrombosis of fetal placental vessels. Therefore, the extent and apparently rapid development of these findings suggests that infection contributed to vascular damage.

The effects of congenital transmission of SARS-CoV-2 remain largely unknown. This study highlights the need for placental and fetal gross and microscopic evaluation, which can help elucidate the pathophysiology of COVID-19.

Appendix Additional information on intrauterine transmission of SARS-CoV-2.
