# Supplementary material for: Intrauterine Transmission of SARS-CoV-2
Source: Emerg Infect Dis. 2021 Feb;27(2):638–41. doi: 10.3201/eid2702.203824 (PMC7853547; doi:10.3201/eid2702.203824)
Supplement: Appendix — Additional information on intrauterine transmission of SARS-CoV-2. [file 20-3824-Techapp-s1.pdf]

Article DOI: https://doi.org/10.3201/eid2702.203824

Intrauterine Transmission of SARS-CoV-2

Appendix

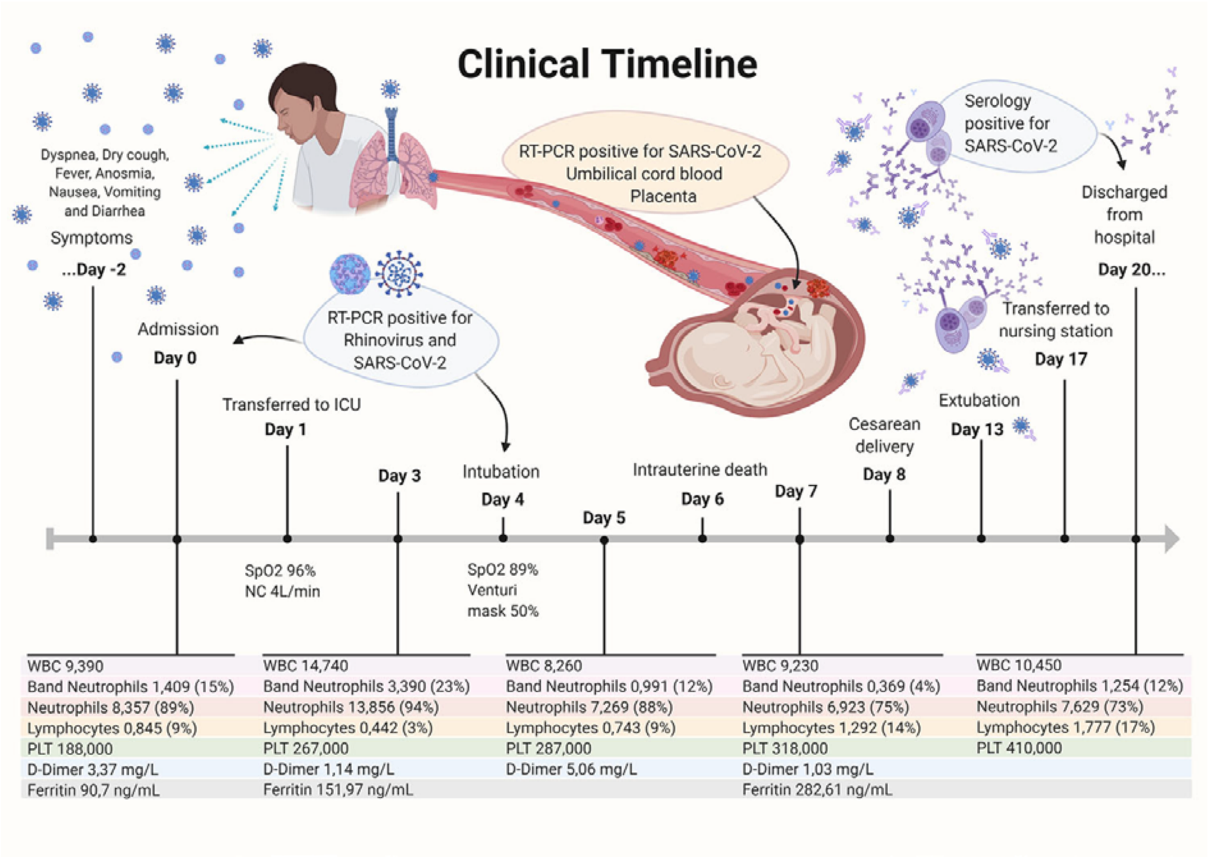

Appendix Figure 1. Timeline of intrauterine transmission of SARS-CoV-2, Brazil, 2020. PCR conducted with XGEN MASTER COVID-19 Kit (Mobius Life Science, Inc, https://mobiuslife.com.br). Image created with BioRender (https://www.getbiorender.com). ICU, intensive care unit; NC, nasal cannula; PLT, platelets; RT-PCR, reverse transcription PCR; SARS-CoV-2, severe acute respiratory syndrome coronavirus 2; SpO2, oxygen saturation; leukocyte, leukocyte.

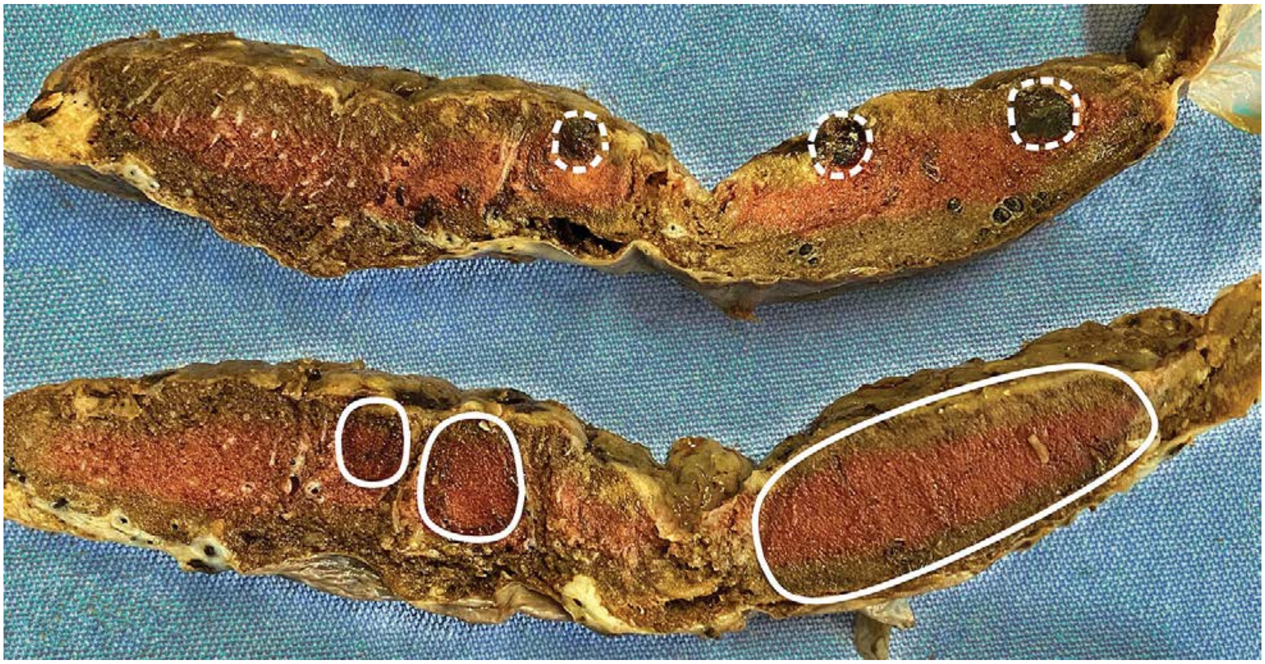

Appendix Figure 2. Placenta of stillborn fetus of a woman with severe acute respiratory syndrome coronavirus 2 infection, Brazil, 2020. Serial sectioning of placental disc shows spongy dark red parenchyma with recent and hemorrhagic nonperipheral infarcts. White circles indicate recent infarcts; dashed white circles indicate hemorrhagic infarcts.

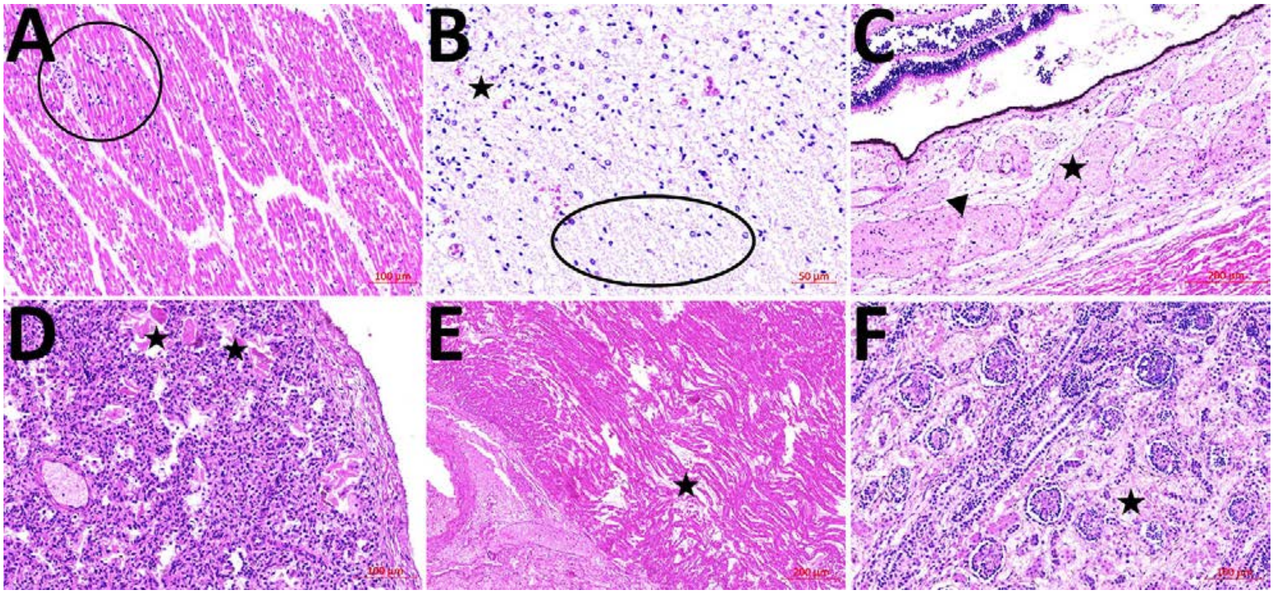

Appendix Figure 3. Histological sections of tissues from a fetus of a woman with severe acute respiratory syndrome coronavirus 2 infection, Brazil, 2020. Tissue stained with hematoxylin and eosin. A) Skeletal muscle with mild interstitial edema. Circled area indicates scattered lymphocytic infiltrate. B) Brain samples show white matter with areas of increased cellularity, suggesting microglial activation. Star

indicates increased cellularity; circled area shows reference area for comparison. C) Cross-section of the eye shows accentuated choroidal edema and congestion. Arrowhead indicates edema; star indicates congestion. D) Lungs show large number of aspirated squamous cells. Stars indicate distal alveolar branches. E) Heart samples show mild interstitial edema and mild lymphocytic infiltrate in the pericardium. Star indicates interstitial edema. F) Kidney shows systemic congestion associated with interstitial hemorrhage. Star indicates congestion.
